# AI-delirium guard: Predictive modeling of postoperative delirium in elderly surgical patients

**DOI:** 10.1371/journal.pone.0322032

**Published:** 2025-06-05

**Authors:** Sri Harsha Boppana, Divyansh Tyagi, Sachin Komati, Sri Lasya Boppana, Ritwik Raj, C. David Mintz

**Affiliations:** 1 Department of Internal Medicine, Nassau University Medical Center, East Meadow, New York, United States of America; 2 Department of Applied Physics, Delhi Technological University, Delhi, India; 3 Department of Computer Science, Florida International University, Miami, Florida, United States of America; 4 Department of Internal Medicine, Alluri Sitarama Raju Academy of Medical Sciences, Eluru, India; 5 Johns Hopkins University Zanvyl Krieger School of Arts and Sciences, Baltimore, Maryland, United States of America; 6 Department of Anesthesiology & Critical Care Medicine, Johns Hopkins School of Medicine, Baltimore, Maryland, United States of America; Hospital Sirio-Libanes, BRAZIL

## Abstract

**Introduction:**

In older patients, postoperative delirium (POD) is a major complication that can result in greater morbidity, longer hospital stays, and higher healthcare expenses. Accurate prediction models for POD can enhance patient outcomes by guiding preventative strategies. This study utilizes advanced machine learning techniques to develop a predictive model for POD using comprehensive perioperative data.

**Methods:**

We examined information from the National Surgical Quality Improvement Program (NSQIP), which included 17,000 patients who were over 65 and undergoing different types of surgery. The dataset included variables such as patient demographics (age, sex), comorbidities (diabetes, cardiovascular diseases, pre-existing dementia), surgical details (type, duration), anesthesia type and dosage, and postoperative outcomes. Categorical variables were encoded numerically, and data standardization was applied to ensure normal distribution. A range of machine learning approaches were assessed such as Decision Trees and Random Forests. Based on the greatest Area Under the Curve (AUC) from Receiver Operating Characteristic (ROC) analysis, the final model was chosen. Hyperparameter tuning was performed using GridSearchCV, optimizing parameters like max_depth, min_child_weight, and gamma for XGBoost model.

**Results:**

The optimized XGBoost model demonstrated superior performance, achieving an AUC of 0.85. Key hyperparameters included min_child_weight = 1, max_depth = 5, gamma = 0.3, subsample = 0.9, colsample_bytree = 0.7, reg_alpha = 0.0007, learning_rate = 0.14, and n_estimators = 123. The model exhibited an accuracy of 0.926, recall of 0.945, precision of 0.934, and an F1-score of 0.939, depicting a higher level of predictive accuracy & balance between sensitivity and specificity.

**Conclusion:**

This study proposes a strong XGBoost-based model to predict POD in older surgical patients, demonstrating the potential of Machine Learning (ML) in clinical risk assessment. With the help of the model’s balanced performance indicators and high accuracy, physicians may identify high-risk patients and promptly execute interventions in clinical settings. Subsequent investigations ought to concentrate on integration into clinical workflows and external validation.

## Introduction

Following surgery, POD is a common and serious complication [1]. Numerous adverse outcomes have been associated with it, such as extended hospital stays, an increased likelihood of being transferred to institutional care upon release, increased readmission rates, functional deterioration, dependency on daily tasks, and cognitive loss [2–9]. Multicomponent non-pharmacologic therapies [10], the Hospital Elder Life Program [11], or perioperative geriatric consults [12] can prevent many cases of POD. There have been reports of successful perioperative strategies that combine targeted delirium prevention practices with delirium risk stratification [13,14], though improving the accuracy of the delirium risk stratification tool that had been utilized in this strategy could improve the distribution of scarce resources to the most vulnerable. Optimizing and automating risk classification processes is crucial since preventative interventions need a significant amount of time and effort from busy professionals [15].

ML-derived risk prediction models had been created to predict delirium in patients who have been hospitalized [16], POD in certain patient categories [17], non-delirium-related intra-op complications [18,19], post-op mortality [20], and other situations range [21]. Existing POD risk prediction models may benefit from the application of the ML approach in several important areas. While age, as well as cognitive impairment, are two well-established risk factors that are frequently included in delirium prediction models [22–24], machine learning (ML) can reveal intricate patterns and interactions between variables within large datasets that are not immediately visible when using more conventional, linear approaches to data analysis [24,25].

Using patient data from the NSQIP, this study evaluates multiple machine learning algorithms with the aim of identifying the most effective POD predicting model. The first step involved preprocessing raw data. Categorical variables such as demographics and anesthesia type were systematically encoded into numerical formats appropriate for machine learning algorithms. Standardization was applied to ensure consistent scaling across all features and minimize bias in model predictions. After comparing the multiple ML model’s performance based on higher AUC from ROC analysis, two models (Random Forest and XGBoost) were identified as the most effective. Further optimization through hyperparameter tuning led to the selection of XGBoost as the algorithm for implementation. The resulting model is a reliable and scalable tool that has the ability to better inform patient care.

## Methods

### Data collection source description

This study draws upon data collected from the NSQIP predominantly U.S.-based, over the period from 2014 to 2020, offering an extensive dataset that includes essential patient demographics and clinical variables. Since this study utilized data from the NSQIP, ethical approval was not necessary. NSQIP is a de-identified, open, publicly accessible database created especially for surgical outcome research and quality improvement.

The use of NSQIP data complies with privacy regulations and ethical guidelines for research involving human participants’ data, eliminating the need for additional ethics review. The NSQIP data set was accessed on 23rd August 2023. The information encompasses key demographic factors like age and sex, along with significant clinical details such as the presence of diabetes, hypertension, cardiovascular conditions, pre-existing dementia, psychiatric disorders, and preoperative delirium. Additionally, the dataset provides insights into the types of surgeries performed, their durations, pain management protocols, anesthesia types and dosages, and overall preoperative health status. A critical focus is placed on the occurrence of postoperative delirium.

### Population criteria variables

The study’s population comprises individuals aged 65 years and older who underwent surgical procedures requiring anesthesia. The key variables analyzed include patient sex, age, inpatient or outpatient status, transfer status, type of anesthesia administered, discharge destination, patient height and weight, surgical specialty, whether the surgery was elective, smoking status, presence of dyspnea, cancer diagnosis, diabetes status, levels of preoperative sodium and albumin, hematocrit levels, whether the procedure was an emergency, surgery duration, presence of renal insufficiency, use of steroids, wound classification, presence of wound infection, and the occurrence of sepsis.

### Datasets

**Fig 1:**
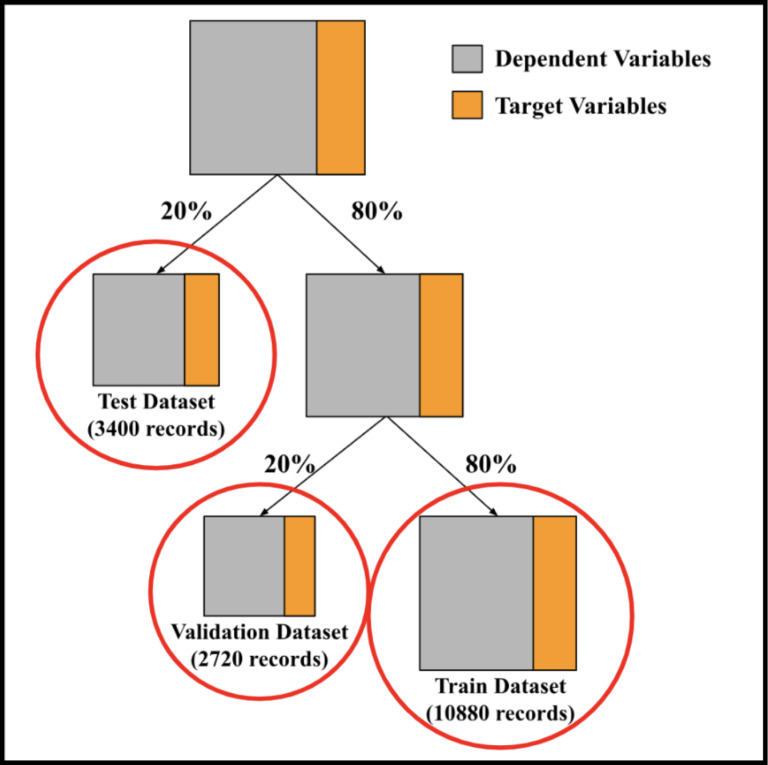
Distribution of data.

This study’s main objective is to analyze the risk factors for POD in light of these characteristics. The dataset, which contains 17,000 records, was divided into two classes: 10,867 records where postoperative delirium was diagnosed, and 6,133 records where it was not. To develop and test predictive models, the data was split into a training set (80percent of total records), a test set (20 percent of the total records), and a validation set (20 percent of the training data), resulting in 10,880 records in the training set, 3,400 in the test set, and 2,720 in the validation set, as illustrated in Fig 1.

In preparing the dataset for analysis, categorical variables were converted into numeric formats, which are necessary for use in most machine learning models. For instance, the variable ‘transfer status’ was encoded into categories such as not transferred, transferred from an acute care hospital, and other relevant categories. Similarly, ‘discharge destination,’ ‘anesthesia type,’ ‘surgical specialty,’ and ‘diabetes status’ were systematically encoded into numerical values. Binary variables, such as sex, inpatient or outpatient status, elective surgery, smoking status, cancer diagnosis, presence of wound infection, steroid use, and emergency status, were also encoded numerically to streamline the analysis.

**Fig 2:**
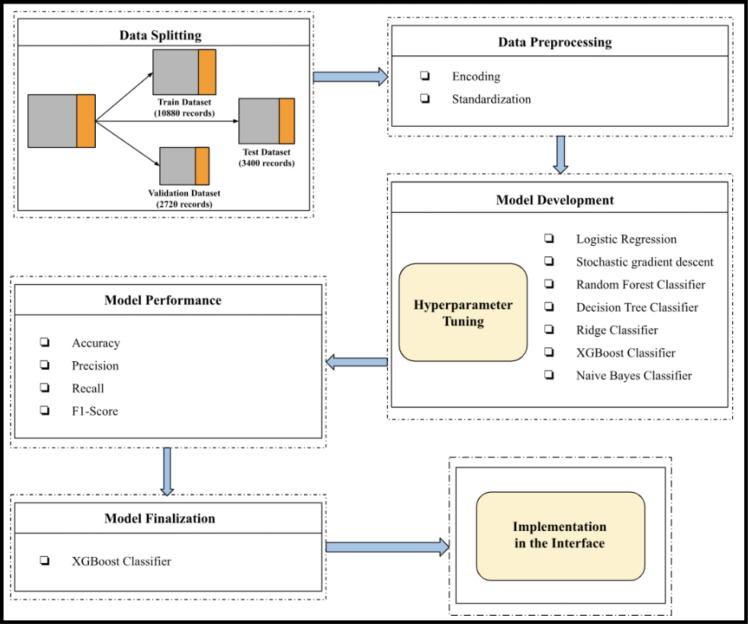
Workflow of the model.

Furthermore, normalization was applied to the dataset to ensure that every feature had a mean of 0 and an SD of 1. This is a crucial step since similar-scale input data reduces the likelihood of biased findings and enhances the performance of many ML algorithms. The process of data preparation, including encoding and standardization, is summarized in the study’s workflow diagrams in Fig 2.

### Data preprocessing

Since many machine learning methods demand numerical inputs, it was required to convert categorical data into numerical representations in order to prepare for machine learning analysis. Specific encoding methods were applied to different categorical variables. For the `transt` variable, patients admitted from home were assigned a value of 0, those transferred from an acute care hospital inpatient setting were coded as 1, from an external emergency department as 2, and other sources of transfer as 3. In the `dischdest` variable, representing discharge destinations, home was coded as 0, other destinations as 1, rehabilitation centers as 2, and skilled care facilities as 3.

The type of anesthesia used, recorded in the `anesthes` variable, was numerically encoded with general anesthesia as 0, MAC/IV sedation as 1, spinal anesthesia as 2, and other forms as 3. Surgical specialties were categorized in the `surgspec` variable, where urology surgery was assigned 0, orthopedics surgery 1, general surgery 2, neurosurgery 3, thoracic surgery 4, head and neck surgery (ENT) 5, vascular surgery 6, plastic surgery 7, cardiac surgery 8, and gynecology surgery 9.

Additionally, the presence and type of diabetes were categorized in the `diabetes` variable, with no diabetes encoded as 0, non-insulin-dependent diabetes as 1, and insulin-dependent diabetes as 2. Dyspnea, reflected in the `dyspnea` variable, was encoded as no dyspnea (0), dyspnea on moderate exertion (1), and dyspnea at rest (2). Wound classification, indicated in the `wndclas` variable, was categorized with clean/contaminated wounds as 0, clean wounds as 1, dirty/infected wounds as 2, and contaminated wounds as 3.

Demographic and procedural variables were also encoded, with `sex` categorized as male (0) and female (1), and `inout` as inpatient (0) and outpatient (1). Binary variables, such as `electsurg`, `smoke`, `discancr`, `wndinf`, `steroid`, `prsepis`, and `emergncy`, were uniformly coded as no (0) and yes (1).

### Standardization

The standardization of data was an essential step to enhance the performance of machine learning models, particularly since different scales among features can lead to biased predictions. This process involved scaling the data so that the mean of each feature was adjusted to 0 and the standard deviation to 1. This transformation ensured that the features were on a common scale, thereby improving the accuracy as well as reliability of the model’s predictions.

### Model development

#### Algorithm selection.

In the development of our predictive model, we evaluated multiple machine learning algorithms to determine the most effective approach for our specific dataset. The algorithms explored included LR, SVM, DT Classifier, Stochastic Gradient Descent, Ridge Classifier, Random Forest Classifier, Extreme Gradient Boosting Classifier, and Naïve Bayes Classifier. Each model was implemented and assessed based on its performance metrics, particularly focusing on its discriminative ability.

**Fig 3:**
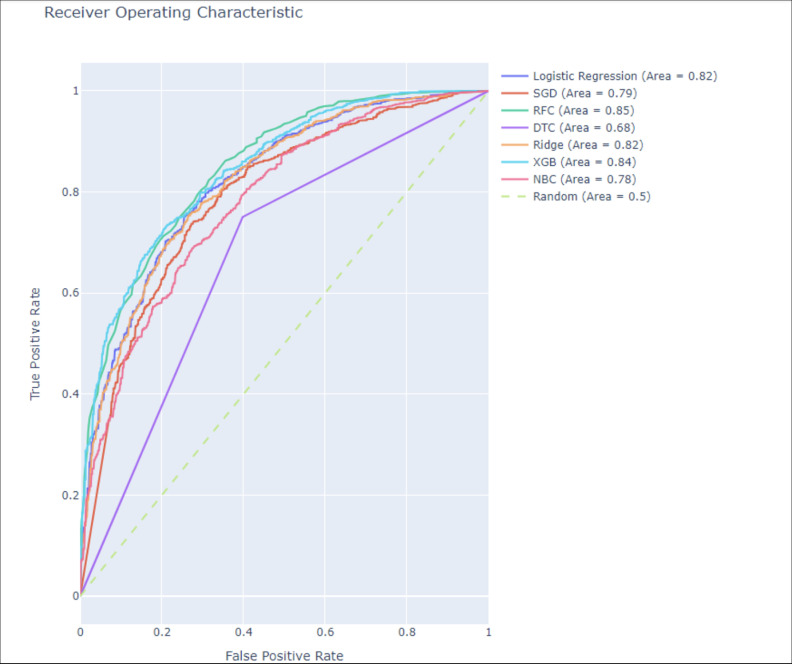
ROC-AUC curve of models implemented.

The ROC curves for these models, as depicted in Fig 3, were analyzed with the AUC serving as the key criterion for model selection. Based on this analysis, the RF Classifier and Extreme Gradient Boosting Classifier were identified as the top-performing models, achieving AUC values of 0.85 and 0.84, respectively. These models were thus selected for further refinement and validation.

Fig 3 shows the ROC curve of the models implemented and the AUC value is used to choose the best model. Among the various models, we have chosen the Random Forest Classifier and Extreme Gradient Boosting Classifier having AUC 0.85 & 0.84 respectively.

#### Hyperparameter tuning.

To further enhance the selected model’s performance, hyperparameter tuning was conducted using GridSearchCV. With this approach, one can perform a thorough search across designated parameter grids and methodically assess various combinations to determine the ideal configurations. A further step in the GridSearchCV process is K-fold cross-validation, which splits the dataset into the k subsets. Each iteration ensures a model performance thorough evaluation across many parameter configurations by setting aside one subset for validation and using the remaining k-1 subsets for training.

For the Extreme Gradient Boosting (XGBoost) model, the optimal hyperparameters identified through this process were as follows: min_child_weight=1, max_depth=5, colsample_bytree=0.7, gamma=0.3, subsample=0.9, learning_rate=0.14, reg_alpha=0.0007, and n_estimators=123. Similarly, for the Random Forest model, the best hyperparameters were found to be max_depth = 40, n_estimators = 174, max_features=‘sqrt’, max_leaf_nodes = 3500, min_samples_leaf=1, and min_samples_split=2. These optimized parameters significantly improved the predictive accuracy and overall performance of the models, validating the effectiveness of our approach.

## Results

### Model performance

The models’ efficacy has been analyzed by utilizing 4 primary classification metrics: F1-score, accuracy, recall, and precision. The cases accurately percentage classified relative to the total number of cases is known as accuracy. Recall assesses the model’s capacity to find out the positive cases, whereas precision measures the positive prediction accuracy. When working with unbalanced datasets, the F1-score offers a comprehensive classification statistic by providing a harmonic mean of precision & recall.

**Fig 4:**
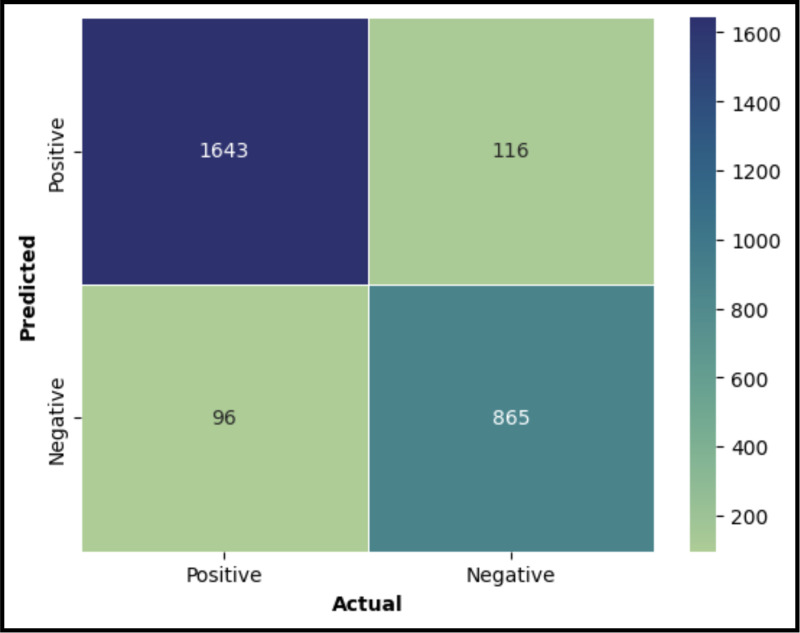
Confusion Matrix for XGBoost Classification Model.

**Fig 5:**
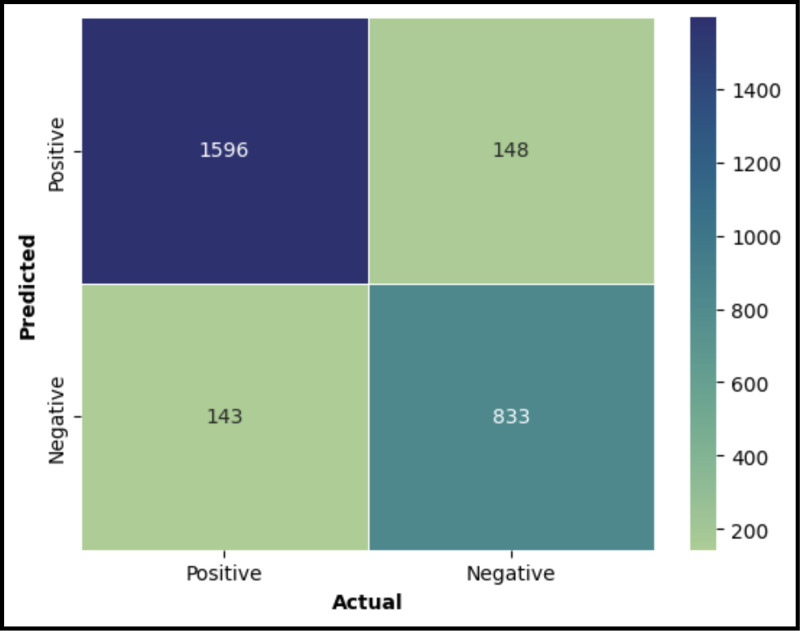
Confusion Matrix for Random Forest Classification Model.

**Fig 6:**
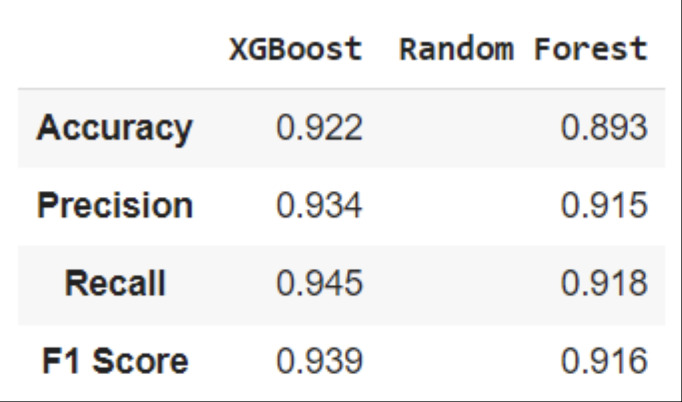
Classification metrics for XGBoost and RF: The table below depicts the values of metrics for XGBoost and Random Forest model.

**Fig 7:**
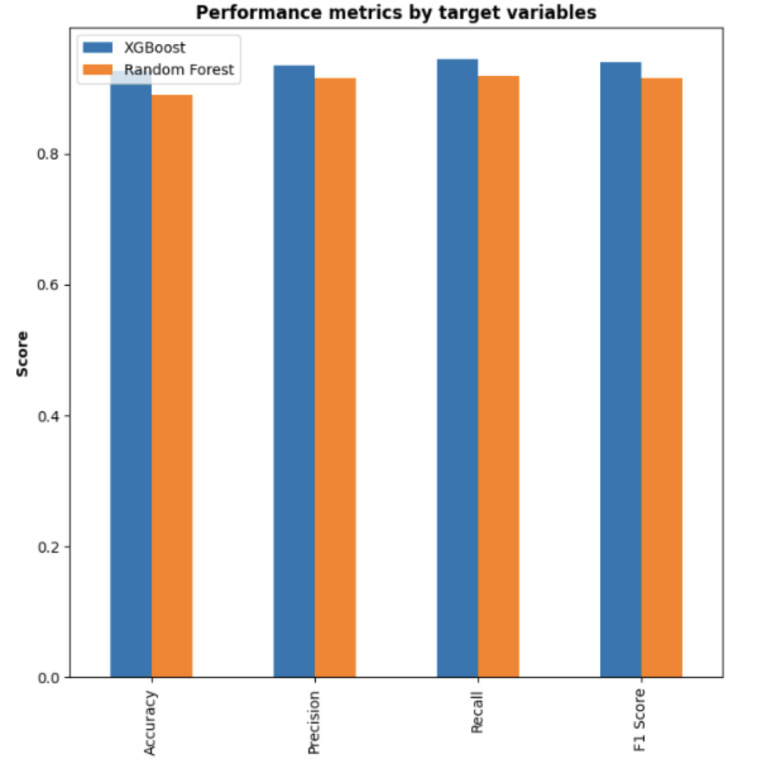
Comparison of both models: It can be understood that the XGBoost outperforms Random Forest in terms of classification metrics. So, we have chosen XGBoost as our final model to be used in the interface. The interface helps the user to predict the possibility of postoperative delirium.

The models were validated using a separate dataset, with the results illustrated in the confusion matrices (Fig 4 for the XGBoost model and Fig 5 for the Random Forest model). These matrices are essential for visualizing the model’s classification outcomes. A summary of the performance metrics for both models is presented in Fig 6. From this comparison (Fig 7), it is evident that the XGBoost model surpasses the Random Forest model in all evaluated metrics. Consequently, XGBoost was chosen as the final model to be integrated into the user interface designed to predict POD risk in elderly patients

### Interface description

The interface is designed to assist healthcare providers in the early detection of post-op delirium, offering quick and accurate risk predictions based on the data entered. The interface is user-friendly, featuring multiple input methods tailored for different data types. For categorical variables, users can select options from drop-down menus, while numerical inputs are entered directly into designated fields. For ease of use, height is entered in separate fields for feet and inches, and weight can be entered in either kilograms or pounds, based on the user’s preference. After all data has been entered, the ‘Predict’ button is pressed to generate a prediction. The interface then displays a message indicating either a high likelihood (“You are likely to experience postoperative delirium after surgery”) or a low likelihood (“You likely will not experience postoperative delirium after surgery”) based on the model’s output.

### Model implementation in the interface

User input data collected through the interface is first compiled into a data frame. Since the data includes both categorical and numerical variables, which have not been standardized, preprocessing is necessary. Categorical data is encoded in the same manner as during the initial model development, ensuring consistency. Numerical data is standardized using parameters saved during the model’s training phase. The processed data is then passed by the trained XGBoost model to generate a prediction. Depending on the outcome, the interface displays a corresponding message: either “You are likely to experience POD after surgery” for a high-risk prediction or “You likely will not experience postoperative delirium after surgery” for a low-risk prediction.

**Interface Link:** https://ai-delirium-guard.streamlit.app

## Discussion

The accurate prediction of POD in elderly surgical patients is a significant clinical challenge, with existing methods primarily reliant on subjective clinical assessments. These traditional approaches, while valuable, often lack the precision needed to consistently identify at-risk patients before the onset of delirium. The findings of our research highlight the potential of advanced ML techniques, particularly the XGBoost algorithm, to significantly enhance the predictive accuracy for POD, offering a more reliable tool for clinical decision-making.

Postoperative delirium has been the subject of extensive research due to its association with increased morbidity, extended hospital stays, and elevated healthcare costs. Traditional methods for predicting POD typically involve clinical scoring systems that assess known risk factors like age, baseline cognitive function, and the presence of comorbidities. Tools like the Confusion Assessment Method (CAM) have been commonly utilized to diagnose delirium postoperatively but are not designed to predict its occurrence beforehand (28).

The utilization of ML models to forecast postoperative outcomes, like POD, has gained popularity recently. For instance, Bishara et al. explored the use of ML algorithms to predict postoperative delirium, finding that models like Random Forests and Gradient Boosting Machines could outperform traditional risk assessments in accuracy. (29) Nevertheless, a lot of this research has been constrained by single-center datasets or small sample numbers, which can limit how broadly the results can be applied.

Machine learning-derived models demonstrated high AUC-ROCs; these models outperformed numerous risk prediction models, especially those designed for postoperative delirium [21,26], and they were on par with other published ML-derived risk prediction models [15,27–29]. Unlike most previously published POD risk prediction methods, which focus on a specific surgical group, this model aims to predict delirium over a wide range of variables [22]. In a comparable non-specific perioperative population from our institution, ML models significantly enhanced discrimination when compared to a basic regression technique [26].

Our study advances this field by utilizing a large, multicenter dataset from the NSQIP that encompasses a wide range of perioperative variables. By employing the XGBoost algorithm, which has been optimized through rigorous hyperparameter tuning, we achieved an AUC of 0.85, a substantial improvement over the AUC values typically reported for logistic regression models used in similar contexts [13]. This performance indicates that the XGBoost model can capture complex interactions between variables that traditional models may miss, offering a more nuanced and accurate risk prediction.

Giving patients who have been at risk for delirium the best care possible requires a lot of resources. Interventions like multicomponent non-pharmacologic nursing care bundles along with time-constrained consultations from physicians like pharmacists and rehabilitation specialists are part of this process. The published guidelines for the prevention of POD [1,14,22,30] served as the paradigm for the care treatments. Preoperative cognitive screenings and avoiding high-risk drugs are advised by the majority of guidelines. The postoperative elements of our approach, such as multicomponent bundles that address early mobility, medication review, and underlying reasons, are in line with postoperative care guidelines [1,14,22,30]. It is essential for the identification of patients who are most likely to have delirium to facilitate their admission into this treatment path. Enhancing the efficacy of delirium screening models could facilitate more efficient resource allocation for high-risk patients, hence improving the overall value of healthcare. The model will next be operationalized through external and prospective validation, and then it will be integrated into the EHR for real-time use. This technique is feasible, as evidenced by earlier descriptions of comparable real-time ML model implementations [31].

Furthermore, while previous studies have focused on general surgical populations, our research specifically targets elderly patients, a demographic particularly vulnerable to delirium due to factors such as frailty, polypharmacy, and multimorbidity [17]. The inclusion of a comprehensive set of perioperative variables, such as anesthesia type, surgical duration, and comorbid conditions, further enhances the model’s applicability in clinical practice.

This conclusion is shown by contrasting our results with a recent investigation by Racine, et al. [32]. The study assessed the ability of five machine learning algorithms to forecast POD in a far smaller subset of surgery patients—560 older adults drawn from an already-existing dataset. Despite employing medical document checks and in-person examinations by skilled interviewers utilizing the CAM [17] to diagnose delirium, machine learning algorithms showed AUC-ROC values of 0.53–0.71, which did not beat the logistic regression model presented in the research. This comparison emphasizes how crucial it is to have a large enough training dataset [33], among other things, in order to perform excellent machine learning studies. Even with our enormous sample size, our results are fairly rare, thus the model’s performance in terms of discrimination, positive predictive value, and calibration would probably improve further with more data and a larger number of occurrences per predictor [33].

In machine learning studies, it is common to criticize reporting model discrimination as the only performance indicator [34], since other performance measures that take occurrence into account might more accurately reflect clinical application & significance. Large positive probability ratios for XGBoost allow for the delivery of resource-intensive therapy to patients who are most at risk of growing delirium. We used thresholds for optimizing sensitivity and positive probability ratio rather than PPV because the former is largely influenced by disease prevalence. Furthermore, compared to the expert clinician regression model, the ML hybrid model that utilizes predictors from the XGBoost feature importance summary performs better. This suggests that ML-based models could be able to identify higher-order interactions between variables that are hard to locate with conventional regression approaches. The majority of the XGBoost-identified predictors, such as age, type of surgery, cognitive impairment, and comorbidities, are in line with previously established POD factors [35–37]. Not often mentioned but crucial to the model are protective factors against delirium such as private insurance, alcohol consumption self-reported history, and a self-reported history of recent falls. These attributes probably point to relationships that the model does not account for. People who own private insurance, for instance, might be better educated and/or have a higher social standing. Individuals who are able to self-report falls may have superior cognitive performance over those who are not. Whilst self-reported alcohol use has been associated with better functional outcomes, such as reduced frailty in females [38] and a lower probability of mobility or arm function restrictions independent of muscle strength in older men [39], alcohol consumption is a known risk factor for delirium [40]. Some authors suggest that the protective effect of moderate alcohol use may be explained by social or lifestyle characteristics that are not included in other components of the ML model [39]. Further research is necessary because the precise mechanisms behind these relationships are unknown.

By utilizing the XGBoost model, clinicians can identify high-risk patients with greater accuracy and tailor interventions accordingly. For example, patients identified as having a higher risk of POD could benefit from enhanced postoperative monitoring, targeted pharmacological management to reduce delirium risk and the implementation of non-pharmacologic strategies such as cognitive stimulation and early mobilization. These interventions, when applied selectively to those most likely to benefit, could decrease the incidence of POD, shorten hospital stays, and lower healthcare costs [41–49].

### Limitations

This study has certain drawbacks that need to be acknowledged, despite its positives. First, while the NSQIP dataset provides a robust foundation for model development, it may not capture all relevant variables that influence POD, such as specific intraoperative events or detailed cognitive assessments. Additionally, the study’s retrospective design, while necessary for model training and validation, may introduce biases related to data quality and completeness.

Second, although the model demonstrated high predictive accuracy in the validation cohort, external validation using data from other institutions is necessary to confirm its generalizability. The model’s performance may vary in different clinical settings, particularly those with different patient demographics, surgical practices, or perioperative care protocols.

Third, even while XGBoost and other machine learning models have a lot to offer in terms of prediction accuracy, their complexity can make them difficult to understand. Clinicians may be hesitant to rely on models that function as “black boxes” without a clear understanding of how predictions are generated. Therefore, efforts to enhance the transparency and interpretability of these models are crucial to their successful adoption in clinical practice.

The NSQIP dataset is limited in its geographical scope and temporal range, and this study employed it for both model training and testing. This raises important questions about the model’s capability to adapt to different clinical settings and whether its predictive accuracy can be maintained across diverse patient populations and healthcare environments.

Moreover, the successful application of this approach relies on the extensive availability, of patient-specific data that are consistent with the variables recorded in NSQIP. However, not all healthcare systems have access to such detailed data, and when these variables are available, their measurement—often binary in nature (e.g., history of stroke)— may vary. These differences could influence the model’s performance and limit its generalizability across different healthcare settings.

### Future directions

To expand on the results of this study, future research should concentrate on a number of important areas. First, external validation of the XGBoost model across diverse patient populations and healthcare settings is essential to ensure its broader applicability. Prospective studies that integrate the model into clinical workflows and evaluate its impact on patient outcomes will also be important for demonstrating its practical utility.

Additionally, further refinement of the model to incorporate real-time data inputs, such as intraoperative monitoring or postoperative cognitive assessments, could enhance its predictive accuracy and clinical relevance. Exploring the integration of other ML approaches, such as deep learning or ensemble models, may also provide additional insights and improve prediction capabilities.

Finally, there is a need for ongoing research into the pathophysiological mechanisms underlying POD, which could inform the development of more targeted and effective interventions. ML models, with their capability for the identification of complex patterns in large datasets, might play an important role in advancing our understanding of these mechanisms and ultimately improving patient care.

To extend the implications of our findings, we propose that an in-depth analysis of the decision-making processes within the XGBoost model could offer novel insights into the underlying pathophysiology of postoperative delirium (POD). By examining the factors and patterns that the model identifies as significant, researchers may uncover previously unrecognized mechanisms contributing to POD. This methodology has promise in filling in knowledge gaps and opening doors for the creation of more specialized and potent treatment approaches.

## Conclusion

This study provides compelling evidence that machine learning, particularly the XGBoost algorithm, can significantly enhance POD prediction in elderly surgical patients. The creation of a dependable, computer-based prediction model is a promising development in perioperative treatment that could lead to better patient outcomes by identifying POD early and preventing it specifically. To ensure this model’s successful integration into clinical practice and maximize its impact on patient care, future research should work to validate and improve it.
